# Supplementary material for: Are carbon emissions trading and green financial instruments synergistic? -Comprehensive quantitative research based on content analysis
Source: PLoS One. 2024 Mar 7;19(3):e0298601. doi: 10.1371/journal.pone.0298601 (PMC10919601; doi:10.1371/journal.pone.0298601)
Supplement: S1 Data — (ZIP) [file pone.0298601.s001.zip › Data/地级市Z_F工作报告.zip!/░▓╗╒/╗┤─╧╩╨/└┤╫╘╣1⁄2╓┌║┼í╛┬φ┐╦╩2╛▌═°í┐_2018.docx]

各位代表：

现在，我代表市人民政府，向大会报告政府工作，请予审议，并请市政协委员和其他列席人员提出意见。

一、过去五年工作回顾

市十五届人大一次会议以来的五年，是淮南发展进程中极不平凡的五年。面对资源型城市转型、经济运行下行、化解过剩产能等巨大压力，全市人民在省委省政府和市委的坚强领导下，坚持以习近平新时代中国特色社会主义思想为指导，积极践行新发展理念，牢牢把握稳中求进工作总基调，攻坚克难、奋力进取，顺利完成“十二五”规划，稳步实施“十三五”规划，全市改革开放和现代化建设取得了新的成就。

——综合实力迈上新台阶。初步核算，2017年地区生产总值1100亿元，年均增长5.4%；固定资产投资1050亿元，年均增长7.8%，完成市人代会确定的“双千亿”目标；财政收入162.3亿元；规上工业增加值365亿元，年均增长4.2%；社会消费品零售总额572亿元，年均增长11.5%。粮食生产“十四连丰”。

——产业结构实现新优化。三次产业结构比由11.2：59.8：29调整为10.8：48.7：40.5，非煤电产业增加值占规上工业比重由19.9%提高至26.8%，民营经济增加值占GDP比重由41.3%提高至46%。

——重大项目取得新突破。中安煤化一体化、商合杭高铁淮南段等加快建设，中国移动（安徽）数据中心一期、安理大新校区、平电三期等建成投用,济祁高速淮南段、凤台淮河二桥、孔李淮河大桥等建成通车,全球最大的水面漂浮式光伏电站并网发电。

——发展活力获得新提升。成功纳入中原经济区、淮河生态经济带、全国老工业基地调整改造规划等国家级平台。淮南经开区成为皖北地区首家国家级经开区。扎实推进615项改革任务，完成或取得阶段性成果595项。国家智慧城市和省级创新型城市试点稳步推进。新增“四上”企业173家。寿县顺利划转并平稳发展，为我市“四化同步”拓展了空间。

——人民生活得到新改善。城镇新增就业25.6万人，农村劳动力转移就业15.9万人。城乡居民收入分别达30065元、11716元，年均分别增长7.7%、9.5%。居民消费价格指数温和上涨。PM10平均浓度下降7%。贫困人口减少12.48万人，贫困发生率下降至2.62%，在打赢精准脱贫攻坚战征程上迈出了坚实步伐。

五年来，我们落实重大部署、紧扣“四张资源牌”，转型步伐愈发坚定。全面落实省市五大发展行动计划，坚持“1235”发展思路，明晰“235”产业转型路径。打优煤电资源牌，依法关闭15对小煤矿，全面迎来现代化大矿井时代。累计生产原煤3.9亿吨、发电量2857亿千瓦时。光伏发电在建装机规模480兆瓦，并网装机容量1000兆瓦。朱集西矿、两个1000千伏特高压工程、6个超临界机组竣工投用。打强数据资源牌，大数据产业基地获批省战新产业基地，入驻关联企业586家，国内首个钱学森智库分中心、大数据展示中心、江淮云、智慧谷建成投用，初步形成大数据存储、交易、应用三大体系。打好科教资源牌，累计投入研发经费72亿元。拥有国家级创新平台7个、国家级科研院所2家、院士工作站10家、高新技术企业78家，新增专业技术及高技能人才6.3万人。打响文化资源牌，推进文化旅游强市建设。成功创建3个国家4A级景区。八公山风景名胜区总体规划获批实施。少儿艺术获评国家公共文化示范项目，一批文艺精品荣获国家奖项。寿州古城被评为中国华侨国际文化交流基地，凤台县荣获中国民间文化艺术之乡称号。旅游总收入累计达333.8亿元，文化产业增加值年均增长10%以上。

五年来，我们坚持稳中求进、推动综合施策，发展基础日趋稳固。出台稳增长系列政策，建立健全调度通报考核机制，经济发展触底回升、逐年向好。坚持“四督四保”机制，开工亿元以上省重点项目450个、竣工233个。完成民间投资、工业投资2054亿元、1690亿元，分别是上个五年的4.6倍、1.9倍。加强与央企、知名民企战略合作，引进内资是上个五年的3倍。成立海关、商检机构。利用外商直接投资、进出口总额分别是上个五年的1.5倍、2倍。合淮同城化加快推进，园区、交通、教育等领域合作共建成效显著。投放产业发展基金21.7亿元。实现直接融资1398.5亿元。金融机构人民币存贷款余额分别迈上2000亿元、1000亿元台阶。发放“税融通”、新型政银担贷款、续贷过桥资金132.4亿元，为企业结构性减税54.9亿元。强化责任担当，为408家中小微企业化解了977个历史遗留问题。

五年来，我们深化改革创新、完善体制机制，发展活力持续释放。供给侧结构性改革深入实施，“去降补”工作成效明显。关闭退出4对煤矿、895万吨产能，妥善安置职工2.2万人。2017年末商品房去化周期14.9个月，两年减少3.1个月。规上农产品加工企业282家，累计实现农产品加工产值265亿元。新增国家地理标志保护产品2个。流转土地172万亩。成功创建安徽淮南（凤台）国家农业科技园。行政审批事项精简52%，涉企收费清单项目减少61%。“三供一业”分离移交稳步推进。在全省率先实施“五证合一”等商事制度改革，新增各类市场主体9.8万家。“营改增”试点改革全面推开，荣获全国百佳国地税合作市级示范区称号。完成党政机关公车改革、不动产统一登记改革。稳妥实施城市管理体制改革。完成8家城市公立医院医改，在全省率先开展按病种收费改革。农村综合改革成效明显，寿县乡镇站所人员管理体制改革走在全省前列。授权有效发明专利1711件，万人有效专利拥有量4.5件。新增中国驰名商标3件。制修订国家标准15项、行业标准70项。建成全省唯一的市级标准化研究院。第八次荣获全国科技进步先进市称号。

五年来，我们统筹城乡建设、推动功能完善，城乡面貌不断改善。城乡建设投资累计突破1000亿元，建成区面积达130平方公里，常住人口城镇化率达63%。持续推进新型城镇化试点，在全省率先启动“多规合一”试点，寿县国家级“多规合一”试点经验全省推广。高新区（山南新区）完成投资428.7亿元，建成项目136个，常住人口达10万人。改造老旧小区128个，建成保障性安居工程5.9万套，惠及群众16.7万人。完成3条主城区铁路下穿改造工程，改造提升老城道路62公里。新增城区道路380万平方米、城市绿地530万平方米。新改建各类管网510公里。推进农村环境整治“三大革命”。建成27个美丽乡镇、211个中心村。2万户农村危房得到改造，82.3万农村人口饮水安全问题得到解决，2472户渔民结束“水上漂”生活。升级改造农村公路1757.3公里。扎实推进“六城同创”。44人荣登“中国好人”榜。依法开展40余项专项整治。征迁拆违2200万平方米。新辟调整公交线路42条，新增公交车782辆，公共自行车成为便民服务新亮点。

五年来，我们狠抓生态建设、守护碧水蓝天，人居环境逐步向好。实施大气污染防治项目1782个，开展燃煤锅炉整治等十余项环保专项行动。非法码头、主城区燃煤锅炉全部拆除，火电企业全部完成脱硫除尘改造。扎实推进全国水生态文明城市试点。在全省率先推行“河长制”。城市污水处理率达93.1%。“十二五”期间污染物总量减排、秸秆禁烧等工作受省通报表彰。国家资源综合利用示范基地加快建设。建成4个生活垃圾处理项目，建筑垃圾处置及再生资源化利用试点经验在全省推广，城市生活垃圾无害化处理率达100%。单位生产总值能耗年均下降3.9%。成功创建焦岗湖国家湿地公园，建成中央公园等7座城市公园。治理采煤沉陷区26平方公里，搬迁居民2.3万户、5.7万人。建立“林长制”，成片造林27万亩，荣获全国绿化模范城市称号。

五年来，我们牢记为民宗旨、推动共建共享，人民得到更多实惠。全市民生支出764.6亿元，占财政支出总量的82.6%。累计实施民生工程50项。严格落实“六个精准”“五个一批”，坚持“六看六确保”，在全省率先实现驻村扶贫工作队和扶贫专干专职化全覆盖。整合扶贫资金15.4亿元，十大工程扎实推进。发放创业担保贷款11.4亿元，实现零就业家庭动态“清零”。新改扩建幼儿园91所、中小学669所，国家义务教育基本均衡发展县区全覆盖。成功举办市第八届运动会。人均公共体育设施面积居全省前列。新改扩建医院24所，社区卫生服务机构城镇人口全覆盖。人口自然增长率7.5‰左右。退休人员养老金、失业保险待遇、城镇低保、农村低保、抚恤补助等标准均大幅提高。市级社会福利保障区、平安城市视频监控系统一期建成投用。全市矛盾纠纷调处率达97%。安全生产、食品药品安全、信访形势总体稳定。市长热线荣获人民网网民留言办理工作10周年贡献奖。国防教育、国防动员、军民融合、双拥创建工作扎实推进。荣获全国人民防空先进城市称号。援藏援疆、地震、气象、档案、民族宗教、地方志、应急管理等工作不断加强，外事侨务、对台、工会、共青团、妇女儿童、老龄、残疾人和红十字等事业取得新进展。

五年来，我们坚持党的领导、推进依法行政，政府作风持续转变。扎实开展党的群众路线教育实践活动、“三严三实”专题教育、“两学一做”学习教育、“讲重作”专题教育和“敢担当、有作为”集中教育。严格遵守党章党规，带头尊法学法守法用法，荣获全国“六五”普法中期先进市称号。在全国率先出台推进依法行政办法，提请审议地方性法规11件，制定政府规章9件，完成重大事项合法性审查696件。主动接受各方监督，市人大代表议案建议、市政协委员提案按时办复率均为100%。从严落实“两个责任”，全面贯彻中央八项规定精神，扎实开展“酒桌办公”等专项整治。“三公”经费年均压缩27%。推进审计监督和制度规范全覆盖，构建“1+X”权力运行监管体系，积极营造风清气正、干事创业的良好政治生态。

各位代表！过去的五年，我们以强烈的使命担当，打赢了遏制经济下滑阻击战，用实干诠释了初心，用实效凝聚了民心。特别是刚刚过去的2017年，是贯彻落实市第十次党代会精神的开局之年，也是本届政府任期的收官之年。我们扎实做好学习宣传贯彻党的十九大精神的各项工作，在政府系统开展“全面学合肥、对标提效能”活动，大力倡导敢闯敢拼不服输的淮河文化，坚持煤与非煤“双轮驱动”不动摇，坚持项目攻坚和招商突破不动摇，坚持主攻山南和提升老城不动摇，坚守脱贫攻坚、安全生产、生态环保、信访稳定、党风廉政五条工作底线，全市经济稳中有进、进中向好。一年来，我们紧盯实体经济发展，出台促进经济平稳发展“30条”、降成本“26条”、支持制造强市“10条”等政策措施，“四送一服”双百工程获省考核优秀等次，市级政务服务综合考评位居全省前三名。我们聚焦项目投资招商，成功举办合肥都市圈城市党政领导第八次会商会，中安煤化一体化项目快速推进，招商引资和项目建设取得近年来最好水平。我们注重统筹协调发展，启动“两治三改”工作，荣获第四届省文明城市称号，进入全国文明城市提名城市行列。我们坚决扛起生态文明建设政治责任，深度谋划采煤沉陷区综合治理，强力推进中央、省环保督察反馈问题整改，完成一水厂取水口上移改造工程，加快补齐生态环保设施欠账，为长远发展打下了坚实的基础。我们全面贯彻精准扶贫、精准脱贫方略，扎实开展“重精准、补短板、促攻坚”专项整改行动，坚决打好脱贫攻坚年度战役。我们以喜迎党的十九大为契机，积极防范化解重大风险，社会大局保持和谐稳定。初步核算，地区生产总值增长7%，固定资产投资增长10%，财政收入增长7.5%，社会消费品零售总额增长11.5%，城乡居民收入分别增长7.5%、9%，较好地完成了十五届人大六次会议确定的各项目标任务。

各位代表！奋斗曲折艰辛，经验弥足珍贵。实践告诉我们，必须把讲政治作为抓发展的根本保障，牢固树立“四个意识”，坚决维护以习近平同志为核心的党中央权威和集中统一领导，不折不扣贯彻落实党中央、省市委各项决策部署。必须把新理念作为行动的先导，坚定不移贯彻新发展理念，以新理念引领新作为，以新作为推动新发展。必须把抓转型作为最紧迫的任务，坚决打破资源依赖惯性思维，持之以恒实施产业转型战略，集中力量加快非煤产业发展。必须把重实干作为成功的法宝，坚持事不避难、依法行政、创新担当，保持永不懈怠的精神状态和一往无前的奋斗姿态。必须把惠民生作为基本的遵循，坚持以人民为中心的发展思想，让发展成果惠及更多的老百姓。

各位代表！过去五年成绩的取得,根本在于以习近平同志为核心的党中央坚强领导，在于习近平新时代中国特色社会主义思想科学指引。五年来，全市人民在省委省政府和市委的坚强领导下，克难攻坚、砥砺奋进，为现代化五大发展美好淮南建设打下了坚实基础。在此,我代表市人民政府向全市干部群众,向人大代表、政协委员,向各民主党派、工商联、无党派人士、人民团体和各界人士,向驻淮部队、武警官兵和公安干警,向所有支持淮南改革发展的同志们、朋友们,表示衷心的感谢!

同时，必须清醒地看到，政府工作还存在一些不足，解决发展不平衡不充分问题还面临不少困难和挑战。主要表现在，一是经济体量较小、增速不快，县域经济、园区经济、民营经济基础薄弱。二是发展质量和效益有待提高，新旧动能转换不快，非煤电产业特别是制造业是明显短板，产业转型升级任务艰巨。三是资源型城市历史包袱沉重，城乡基础设施、公共服务欠账较多。四是民生领域还有不少短板，脱贫攻坚和环境整治任务艰巨，教育、医疗、养老等方面面临不少难题。五是少数干部适应新常态、推动新发展能力不足，不敢担当、不愿作为的现象不同程度存在。对此，我们将采取切实措施，认真加以解决。

二、奋力开创现代化五大发展美好淮南建设新局面

党的十九大作出了“中国特色社会主义进入新时代”的重大判断，吹响了夺取新时代中国特色社会主义伟大胜利的前进号角。市委十届四次全会作出了“三个同步”的战略部署，科学规划了到本世纪中叶的宏伟蓝图。当前，我市经济正处在转变发展方式、优化经济结构、转换增长动力的攻关期，进入转型提速高质量发展阶段。新时代新淮南仍处于重要战略机遇期，深化改革的红利加速释放，创新驱动的活力持续迸发，全面开放的舞台愈加广阔，全市人民对美好生活的向往更凝聚起团结奋进的深厚力量。这些都为我们观大势、谋全局、干实事，紧密对接“两个一百年”奋斗目标，破解发展不平衡不充分这个最大短板，实现富民强市、转型发展这个最大任务，奋力走好新时代的长征路指明了新方向、增添了新动力。

今后五年工作的总体思路是：全面贯彻落实党的十九大精神和习近平总书记视察安徽重要讲话精神，以习近平新时代中国特色社会主义思想为指导，坚持以人民为中心的发展思想，坚持稳中求进工作总基调，坚持新发展理念，紧扣社会主要矛盾变化，按照高质量发展要求，统筹推进“五位一体”总体布局和协调推进“四个全面”战略布局，坚持以供给侧结构性改革为主线，坚持以五大发展行动计划为总抓手，坚持“1235”发展思路，实施八大战略，建设六个强市，打好三大攻坚战，打造国家级现代化绿色能源基地、国家级资源型城市可持续发展示范区、全国文化旅游名市、国家创新型城市，坚定地在践行新发展理念中闯出资源型城市转型发展的新路，决胜全面建成小康社会，加快建设现代化五大发展美好淮南。
根据市第十次党代会和市委十届四次全会提出的战略目标，今后五年全市发展的总体目标是：

——经济发展更加均衡，综合实力进一步增强。全面落实市委“三个同步”战略部署，主要指标增幅达到全省平均水平，县区园区对经济贡献率不断提高。实现现行标准下农村贫困人口全部脱贫，与全国同步全面建成小康社会。

——经济体系更加完善，创新能力进一步跃升。积极创建国家级接续替代产业示范市，民营经济增加值占GDP比重、非煤电产业增加值占规上工业增加值比重分别提高4个、10个百分点。战新产业、高新技术产业加快成长，科技进步贡献率达55%，现代化经济体系建设成效明显。

——区域发展更加协调，城乡功能进一步完善。新型城镇化水平明显提高，全市常住人口城镇化率达65%。城乡融合发展的体制机制不断健全，“一主一副四组团”城市空间格局基本形成。

——人与自然更加和谐，生态质量进一步改善。生态文明制度体系基本确立，突出环境问题得到有效防治，采煤沉陷区得到有效治理，“三湖四河”岸线和湿地得到有效保护，努力建成山水林城融为一体、宜居宜业宜学宜游的生态文明城市。

——改革开放更加深入，经济外向度进一步扩大。重点领域和关键环节改革走在全省前列。积极融入国家“三大战略”，与合肥都市圈城市深度融合，大通道、大平台、大通关基本实现。

——保障体系更加健全，人民获得感进一步提升。居民收入增长和经济增长同步，劳动报酬提高和劳动生产率提高同步。平安淮南、健康淮南、体育强市建设取得新进展。深入推进“六城同创”，健全现代公共服务体系，成功创建全国文明城市。

各位代表！党的十九大明确提出“支持资源型地区经济转型发展”。对淮南来说，转型发展是必由之路。未来五年乃至更长一个时期，必须用新时代新淮南新的历史方位凝聚力量，做好煤电资源、非煤产业、山水生态三篇文章，着力振兴淮南经济，再创新时代新辉煌。今后五年的主要任务是：

一是坚持以创新驱动转型，着力建设现代化经济体系。把提高供给体系质量作为主攻方向，推动经济发展质量变革、效率变革、动力变革，加快建设实体经济、科技创新、现代金融、人力资源协同发展的产业体系。深化供给侧结构性改革，在重点改革方面取得重大突破，建立新制度、培育新要素、形成新市场、发展新产业、壮大新主体。打造科教强市、数据强市，主动融入省“四个一”创新主平台，积极承接合肥综合性国家科学中心成果转移转化，推动信息基础设施提速升级、共建共享，争创国家新型智慧城市标杆市、国家双创示范基地，建成在全国具有重要影响的大数据中心。以“全创改”建设为载体，支持各类科技研发平台建设，加快产学研深度融合，实现创新链与产业链融合。扎实推动“三重一创”建设，打造4个牵动性强的战新基地，支持基础较好的县区创建国家创新型县区。实施工业强市战略，做精做优煤电化产业链，推动煤电化一体化发展，打造煤电、煤化工两个“千亿级”产业基地；做大做强非煤产业群，系统推进制造强市建设，实施接续替代产业培育行动计划、高新技术企业和创新型企业倍增计划、质量品牌升级工程，培育一批具有核心竞争力的产品、企业、产业、基地。实行更加积极、更加开放、更加有效的人才政策，加快市校深度融合发展，打造人才强市。

二是坚持以协调促进转型，着力提升城市品质。坚持“一尊重、五统筹”理念，注重长远发展，注重减量集约，注重生态保护，注重多规合一，打造“楚风汉韵、能源之都”城市品牌。实施区域协调发展战略，统筹好空间布局、产业布局和人口布局，支持田家庵、大通、山南新区融合发展，打造城市主城区，建设行政文化中心、现代商贸物流综合区、战新产业集聚区、先进制造业基地；支持谢家集、八公山打造城市副中心，统筹推进老工业区整体搬迁改造，重点发展文化旅游产业；支持潘集建设现代化绿色能源基地；支持寿县打造“南工北旅生态县”，推动新桥国际产业园与合肥经开区一体发展；支持凤台建成全省一流经济强县；支持毛集建成淮河之畔一流小城市。坚持“主攻山南、提升老城”，全力以赴聚项目、聚人气，建成山南新区核心区；以“两治三改”为主抓手，以功能提升为主方向，制定城市交通堵点治理规划，实施一批利民便民工程，大力推进老城区改造。健全市矿联席会议机制，推动市矿融合发展。实施乡村振兴战略，强化乡村规划管理，加强美丽乡村、特色小镇、少数民族特色村寨建设，推进农业产业园、科技园、创业园融合发展，加快建设特色精品农业大市、生态有机农业强市，让农业更绿、农村更美、农民更富。

三是坚持以绿色引领转型，着力建设生态文明美丽淮南。淮南山水资源丰富，千里淮河穿城而过，城市绿肺舜耕山横贯东西，上窑山、高塘湖山水一色，寿州古城、八公山、焦岗湖、瓦埠湖遥相呼应，35万亩采煤沉陷区水面镶嵌其间，这是淮南得天独厚的资源禀赋。我们必须树立和践行绿水青山就是金山银山的理念，坚持环境友好导向，统筹山水林田湖草系统治理，加强生态保护红线、永久基本农田、城镇开发边界控制线管控，争创国家森林城市。依托独特的山水资源、人文资源，创建寿州古城、八公山景区、焦岗湖景区国家5A级景区，构建“T”型生态文化旅游长廊，建设文旅强市。探索采煤沉陷区资源化利用新路，把沉陷区变成生态区、开发区、旅游区，打造国家采煤沉陷区综合治理示范城市。建设综合康养产业区，打造养老养生宜居之城。坚持资源节约优先，建立健全绿色低碳循环发展经济体系，壮大节能环保产业、清洁生产产业、清洁能源产业，用3-5年时间打造煤矿开发伴生资源综合利用百亿产业。积极调整产业结构、能源结构、运输结构，推动绿色产品、绿色工厂、绿色园区和绿色供应链的全面发展，能耗和水资源消耗、建设用地、碳排放总量得到有效控制。大力实施环境保护“五个一”行动，坚决打赢污染防治攻坚战，促进资源开发利用与经济社会协调发展。

四是坚持以开放助推转型，着力形成全面开放新格局。用好两个市场、两种资源，积极引进来、走出去。大力承接合肥长三角世界级城市群副中心辐射，打造长三角地区能源基地及新型化工产业基地。落实省“三个联动”部署，依托合肥都市圈加速合淮同城化，加快合淮工业走廊、新桥空港经济示范区淮南联动发展区建设，打造辐射沿淮、撬动皖北崛起的重要增长极。发挥县区园区主力军、主战场、主阵地作用，推动县域经济振兴发展，重点打造“3+2”园区平台，支持淮南经开区、淮南农场、大通开发区融合发展。全面参与淮河生态经济带开发，科学规划利用淮河岸线资源，全面开展引江济淮工程淮南段建设，为淮河通江达海开辟新的黄金水道。建设交通强市，构筑立体综合交通，深度谋划建设大通道、大物流园区，推进皖北城际铁路、沿淮高速公路、淮南港七大港区及物流园区、淮河淮南境内行蓄洪区调整改造，以及瓦埠湖大桥等10座跨河（湖）桥梁隧道重大基础设施建设，构建“一纵两横”高铁网、“两纵三横”高速公路网、“四环七通道”干线路网的交通新格局。创优“四最”营商环境，营造“服务企业、尊重企业家”的浓厚氛围，构建亲清新型政商关系，形成投资贸易自由化便利化新机制。

五是坚持以共享保障转型，着力打造人民满意的幸福城市。始终把人民对美好生活的向往作为奋斗目标，确保全面建成小康社会一个不能少，共同富裕路上一个不能掉队。以收入结构调整和公共产品供给为突破口，加快形成政府主导、覆盖城乡、可持续的基本公共服务体系。拓宽居民增收渠道，逐步提高城乡居民收入水平。实施健康淮南战略，争创全国食品药品安全城市。创新社会治理体系，深化安全生产领域改革发展，打造共建共治共享的社会治理格局。实施军民融合发展战略，创建全国双拥模范城。坚持民生为本、脱贫为先、平安为基，在“七有”领域上不断取得新进展，使人民群众获得感、幸福感、安全感更加充实、更有保障、更可持续。

各位代表！规划未来是为了创造未来。在市委的坚强领导下，只要我们团结带领390万勤劳智慧的淮南人民坚定信心、开拓奋进，继续发扬淮河文化中敢闯敢拼不服输的精神，就一定能开创更加美好的明天！

三、2018年重点工作任务

今年是全面贯彻落实党的十九大精神的开局之年，是改革开放40周年，是决胜全面建成小康社会、实施“十三五”规划承上启下的关键一年，也是新一届政府的开局之年。今年经济社会发展的主要预期目标是：地区生产总值增长7%以上，固定资产投资增长9%以上，财政收入增长7%，社会消费品零售总额增长10%，城乡居民收入分别增长7.5%、8.5%，城镇调查失业率控制在5.3%以内，供给侧结构性改革取得实质性进展，金融财政风险有序有效防控，节能减排完成省下达的目标任务。

重点抓好以下八个方面工作:

（一）聚力改革创新，激发实体经济发展活力。把发展经济的着力点更多放在实体经济上，大力实施民营经济提升工程，培育壮大一批竞争力强的实体企业。全年新增民营企业5000家、个体工商户1.3万家。深化供给侧结构性改革。完成煤炭行业去产能任务，妥善分流安置职工。研究制定去产能煤矿关闭退出闲置资源综合利用方案。加强开发用地和商品房供应管理，促进房地产市场平稳健康发展。设立地方金融监管局，健全金融风险预警处置机制。开展“四清四实”专项整治，健全规范政府举债融资机制。引导金融机构回归本源，加大对实体经济支持力度。支持淮矿集团整体上市，新增上市挂牌企业20家，实现直接融资200亿元。积极推进增量配电业务改革试点。聚焦政策执行程序较为繁琐、优惠政策知晓率不高等政策执行障碍，继续清理涉企收费，降低城镇土地使用税征收标准，打通降成本优惠政策“最后一公里”。常态化开展“四送一服”双百工程，畅通企业问题受理渠道，建立限时反馈、限时督办等机制，营造支持民营企业、本土企业发展的良好环境。推进一批重大改革事项。深化“放管服”、商事制度改革，统一规范行政许可和公共服务事项。搭建“互联网+政务服务”全市“一张网”，探索实施“一次都不跑”事项。强化国资国企监管。基本完成“三供一业”分离移交。深化户籍制度改革。落实环保监察监测体制改革任务。开展第四次全国经济普查、第三次全国土地调查、第二次全国污染源普查，做好打基础、管长远的工作。统筹推进其他各领域改革。实施创新驱动发展战略。积极争创国家级创新型试点城市。完善“1+3+8”科技孵化器体系，新增省级科技孵化器2家，培育省级工程技术研究中心2家。申请专利2500件。推进质量品牌升级工程，培育国家地理标志保护产品1个。建设技工大市，新增高技能人才5000人。

（二）聚力开放合作，扩大有效投入。发挥投资对优化供给结构的关键性作用，树立“项目即发展”理念，按照“六个一批”要求，统筹推进项目、投资、招商“三位一体”突破。抓好大招商。继续开展招商引资突破年活动。修订招商引资扶持政策，完善招商引资考核办法，确保引进内资、亿元以上省外项目资金平稳较快增长。实施外贸主体培育壮大工程，进出口总额增长10%。落实外商投资准入前国民待遇加负面清单管理制度，实际到位外商直接投资增长5%。进行大建设。继续开展项目建设攻坚年活动。开工建设引江济淮工程淮南段、正峡段行洪区调整和建设工程、直接金融基地等130个亿元以上重点项目，加快商合杭高铁淮南段及3个站点、光电产业园等150个亿元以上重点项目建设，建成潘集选煤厂、中储粮淮南直属库等45个亿元以上重点项目。依法稳妥处理项目建设中的遗留问题。促进大合作。坚定不移推进合淮同城化，主动融入合肥综合性国家科学中心、合肥滨湖科学城、合芜蚌自主创新示范区，积极引进工程研究中心、技术创新中心、制造业创新中心和新型研发机构，依托园区合作共建再上一批产业项目。营造大环境。坚持项目层级调度、现场调度，成立市大建办，创新项目谋划推进机制，致力解决项目谋划浅、前期长、推进慢等问题。规范政府投资管理。激发民间投资活力。调整完善集体土地地上附着物、国有土地上房屋征收补偿政策。加快开发区体制机制创新，建立健全开发区综合评价考核体系，支持县区发展园区经济。推动“3+2”园区错位发展，每年均超前建设1-2平方公里基础设施，全年完成投资235亿元。研究出台省级以上开发区工业项目行政事业“零收费”政策，支持园区在法定权限内灵活制定招商引资优惠政策。

（三）聚力非煤产业，推动产业结构优化升级。坚持工业强市战略不动摇，壮大煤电家底，主攻非煤电产业，规上工业增加值增长8%。推动新型工业向中高端迈进。实施规上工业企业培育三年行动计划，每年净增规上工业企业100家。推动“两化融合”，促进“互联网+先进制造业”发展，建设谢家集高端金属制造产业园、大通智创智能装备制造产业园，建成蓝讯电子等60个制造业项目，确保中安煤化一体化项目实现中交。大力发展智慧经济，建设安徽大数据交易中心，谋划建设淮南大数据加工场。战新产业产值和高新技术产业增加值分别增长20%、15%。实施传统产业改造提升计划。建设煤矸石、粉煤灰等煤系固废物综合开发利用项目。全年完成工业投资350亿元，其中技改投资240亿元。推动现代服务业向品质化跃升。增强消费对经济发展的基础性作用，新增限上企业50家。实施现代服务业集聚发展工程，规划建设服务业集聚区2个以上。推动城市核心商圈转型升级、错位发展，建成运营田家庵万达广场、新城吾悦广场等项目。推进城区菜市场改造升级、加油站点建设。完善社区商业网点布局，构建城区15分钟便民生活圈。推动文化旅游业向深层次融合。实施文旅强市“五个一批”工程，加快景区配套基础设施建设。加快安徽楚文化博物馆、毛集花田花海旅游区等8个文旅项目建设。扎实推进国家5A级景区创建，支持寿县古城墙申报世界文化遗产、安丰塘申报全球重要农业文化遗产。深入实施文化惠民工程，积极创建中国成语典故之城。

（四）聚力乡村振兴，推进农业农村现代化。淮南既是工矿大市，也是名副其实的农业大市，必须坚持农业农村优先发展。发展现代农业。坚持质量兴农、绿色兴农，推动农业供给侧结构性改革，实施农产品加工业转型升级、龙头企业培育、农产品品牌创建等农业产业化五大工程。支持休闲农业和乡村旅游健康发展，壮大新产业新业态新模式。加强耕地保护，粮食总产稳定在260万吨以上。推进承包地“三权分置”，完成50个村“三变”改革试点任务。推广“龙头企业+基地+合作社+农户”模式。新增家庭农场50家。开工建设4个农产品加工园及物流园，规上农产品加工业产值增长6%以上。支持凤台争创国家农业高新技术产业示范区。健全农业社会化服务体系，推进粮食产后服务中心和“智慧粮食”仓储信息化建设。深化供销合作社综合改革，增强为农服务能力。建设美丽乡村。基本完成乡镇驻地的改造提升，建设30个市级以上中心村。完成农村存量危房改造。持续推进农村环境“三大革命”，完成5万户农厕、全市乡镇驻地和中小学校旱厕改造，加快33个乡镇生活污水设施建设。加强农田水利基本建设。健全美丽乡村建设长效管护机制。提升农村基本公共服务水平。今年集中力量抓好五项建设的巩固提升：一是农村电商全覆盖巩固提升，丰富完善乡村网点服务功能。二是农村饮水安全巩固提升，全面解决贫困村和贫困户饮水安全问题。三是农村道路建设巩固提升，提升改造农村公路346条、423公里。四是农村电网改造巩固提升，实施新一轮农网改造升级工程。五是农村义务教育巩固提升，完成“全面改薄”年度任务，推动城乡义务教育一体化发展。

（五）聚力统筹协调，提高城乡建设水平。引导“住建规划下乡、水利林业进城”，让乡村与城镇、自然与人文各美其美、美美与共。促进城乡规划建设统筹协调。统筹考虑城市产业布局、城镇发展、人口规模、基础设施建设，加快城市总体规划修编。统筹要素资源、坚持规划先行，推动主城区与副中心、各组团联动发展。统筹做好公路、铁路、水运等重大基础设施规划，明确建设时序，保障重点建设。促进新区建设和老城改造统筹协调。坚持以“先进、长远、超前、留白”的理念引领新区建设，完成山南新区总体城市设计及六大导则编制。支持高新区升级建成国家级高新区。新开工山南新区综合医院等12个项目，推进市级老年公寓、商业综合体等50个项目建设，竣工3所学校、市文化艺术中心等9个项目。按照“密路网、小街区”的理念，推进老城区“两治三改”工作，织补各类城市功能。开工建设保障性安居工程5190套、基本建成1877套。新改建各类管网135公里。启动全国文明城市创建工作。推进智慧城管建设，有效解决城区停车、环卫、占道经营等难题。促进城市对内对外交通统筹协调。畅通城市内循环，开工建设过舜耕山西支路、学院路铁路下穿工程，加快淮南站、十涧湖东路等改造，完成11条道路大中修，淮上淮河大桥建成通车。加快城市大通道建设，开工建设合霍阜高速淮南段，加快G206洛河至曹庵段一级公路改扩建、山南新区至新桥机场快速通道、凤台至毛集快速通道等前期工作。推进现代化新型淮南港建设，制定淮河岸线开发保护计划。开工建设5个公交枢纽站场，开展农客班线公交化改造。创建省级公交示范城。

（六）聚力环境治理，坚决打好污染防治攻坚战。只有恢复绿水青山，才能使绿水青山变成金山银山。不断巩固环保督察反馈问题整改成果，为群众提供更多优质生态产品。实施蓝天行动。坚持铁腕管理，落实环卫湿式保洁，健全完善“数字渣土”平台。坚决落实重污染天气预案，开展秸秆综合利用提升行动，建成大气环境监测系统。实施碧水行动。深入落实“水十条”，全面落实“河长制”，确保水质持续稳定达标。启动农村黑臭水体排查治理，城市建成区黑臭水体消除率达80％。完成第一污水处理厂二期扩建工程、洞化截洪沟等5条水系治理。严厉打击非法采砂行为。加强饮用水水源地保护和城市应急备用水源建设。实施净土行动。全面落实“土十条”。抓好土壤和农业面源污染防治工作，开展集中养殖粪污资源化利用行动。推进生活垃圾、餐厨垃圾无害化处理，完善建筑垃圾再生资源利用体系。实施绿地行动。全面推行“林长制”，人工造林1.5万亩。新增和提升绿地面积350万平方米。编制国家采煤沉陷区综合治理示范城市创建规划、计划。加快实施世行贷款采煤沉陷区项目，谋划实施淮西湖、十涧湖等综合治理项目。新开工沉陷区安置房3200套、搬迁入住3500户。

（七）聚力精准施策，坚决打好脱贫攻坚战。保证现行标准下的脱贫质量，既不降低标准，也不吊高胃口，瞄准特定贫困群众精准帮扶，确保完成省下达的贫困人口脱贫、贫困村出列和寿县贫困县摘帽年度任务。用足政策。加大产业、就业、健康、教育、金融等扶贫政策宣传，推动政策精准落实到村到户到人。注重扶贫同扶志、扶智相结合，深入推进脱贫攻坚十大工程，提升基层基本公共服务水平，激发贫困人口内生动力。做实产业。全面推广“四带一自”产业扶贫模式，实现产业扶贫全覆盖。加快推进行蓄洪区脱贫攻坚工作。支持少数民族和民族聚居地区经济社会优先发展。完善机制。开展脱贫攻坚作风建设年活动。充分发挥考核的指挥棒作用，坚持正向激励，落实“一票否决”制。继续实施定点帮扶、单位包村、干部包户和驻村扶贫工作队制度。完善贫困户动态管理机制，构建稳定脱贫、防范返贫的有效机制。

（八）聚力社会建设，持续增强群众获得感。针对人民群众关心的问题精准施策，尽力而为、量力而行，从小处着手、以小见大，高标准实施好省级民生工程。办好几件惠民实事。在全市公立医院推行居民健康一卡通。安排文化惠民送戏下乡1000场、免费公益电影农村放映1万场。建成3个社区多功能运动场、20条全民健身苑。完成7个县级以上集中式饮用水水源地环境整治。建成舜耕山南侧停车场、老市政府停车场。完成舜耕山湿地公园、西部城区和平广场（公园）建设。优先发展教育事业。制定教育提升行动计划，不断提高教育质量，着力解决中小学生课外负担重、“择校热”、“大班额”等突出问题。实施第三期学前教育行动计划，新改扩建公办幼儿园10所。启动县域内义务教育优质均衡创建。实施高中阶段教育普及攻坚计划。保障特殊群体受教育权益。加快职教园区建设，支持在淮高校建设创业孵化基地和校企合作基地。支持和规范社会力量兴办教育，扩大多样化优质教育供给。提高就业质量。落实新一轮促进就业创业政策，争创省级农民工返乡创业示范园。推进就业扶贫驿站建设，新增发放创业担保贷款2亿元。全年城镇新增就业4.2万人，农村劳动力转移就业3.7万人。加强社会保障体系建设。全面实施全民参保计划，积极推进法定人群社会保险全覆盖。统筹城乡社会救助体系。深入开展工伤残疾预防工作。推进医养结合，加快养老产业发展。推进健康淮南建设。持续深化医药卫生体制改革，加快推进市传染病医院改造、市妇幼保健院新院、残疾人康复中心等项目建设。加大对乡镇卫生院建设的支持力度。打造城市15分钟体育健身圈。创建省级食品药品安全城市。建设平安淮南。推动社会治理重心向基层下移，加快县级矛盾纠纷联合调处中心和四级综治中心建设。完成村（居）民委员会换届。健全立体化、信息化社会治安防控体系，依法打击各类违法犯罪活动。严守安全生产底线，强化应急管理，开展安全保障监测体系建设行动和地质灾害防治行动。加强军地协调，深入推进国防动员、国防教育和双拥工作，形成军民融合深度发展格局。支持工会、共青团、妇联等人民团体更好发挥桥梁纽带作用，做好地震、气象、档案、外事侨务、对台、妇女儿童、全民科学素质、宗教和机关事务管理等工作，加快人民防空、广播电视、地方志、老龄、残疾人和红十字等各项事业发展。

四、不忘初心、牢记使命，全面加强政府自身建设

我们的初心和使命就是为淮南人民谋幸福，为淮南发展创辉煌。新时代要有新气象，更要有新作为。我们将落实好服务和监管双重责任，建设人民满意的服务型政府。

提升学习力，用习近平新时代中国特色社会主义思想武装头脑。坚持党对一切工作的领导，全面贯彻习近平新时代中国特色社会主义思想，树牢“四个意识”，坚定“四个自信”，坚决做到“五个纯粹”。推进“两学一做”学习教育常态化制度化，开展“不忘初心、牢记使命”主题教育，在学思践悟中增强学习本领、政治领导本领、改革创新本领、科学发展本领、依法执政本领、群众工作本领、狠抓落实本领和驾驭风险本领。

提升服务力，不断增强群众观念和群众感情。把人民放在心中最高位置，自觉反对形式主义、官僚主义，凡是群众反映强烈的问题都严肃认真对待，凡是损害群众利益的行为都坚决纠正。坚持问政于民、问需于民、问计于民，千方百计为群众办好事、办实事、解难事，始终保持与人民群众的血肉联系。

提升执行力，以钉钉子精神做实做细做好各项工作。深入开展“全面学合肥、对标提效能”活动，创新理念、健全机制。推行一线工作法，敢于直面问题，敢于较真碰硬。完善政绩考核评价体系，建立激励机制和容错纠错机制，为干部干事创业撑腰鼓劲。崇尚实干、力戒空谈、精准发力，强化目标管理、闭环管理、督查问效，确保中央和省市委决策部署落地生根。

提升公信力，建设法治政府和诚信政府。依法全面履行政府职能，认真开展“七五”普法，落实重大行政决策公众参与程序规定，推进重大事项合法性审查、政府法律顾问全覆盖。自觉接受人大法律监督和政协民主监督，认真办理人大代表议案建议和政协委员提案。完善政府守信践诺机制，坚持按法规政策办事，按程序流程办事，按法律的主旨精神创新担当办事。全面推进政务公开和电子政务工作，让权力在阳光下运行。

提升约束力，始终保持廉洁本色。把纪律和规矩挺在前面，认真落实“一岗双责”和“两个责任”，深入贯彻中央八项规定精神及实施细则，让政府系统党员干部知敬畏、存戒惧、守底线。坚持有腐必反、有贪必肃，加强政府内部层级监督和审计监督，推动干部清正、政府清廉、政治清明。

各位代表！新时代催人奋进，新使命激荡人心。让我们更加紧密地团结在以习近平同志为核心的党中央周围，在市委的坚强领导下，锐意进取、实干兴淮，勇做新时代的坚定者、奋进者、搏击者，为决胜全面建成小康社会，加快建设现代化五大发展美好淮南而努力奋斗！
